# Community engagement strategies for preventing recurrent Nipah virus outbreaks in Bangladesh and India: adapting a framework for outbreak preparedness and response

**DOI:** 10.7189/jogh.16.03024

**Published:** 2026-08-17

**Authors:** Shuma Banik, Md Zakiul Hassan, Bipin Adhikari

**Affiliations:** 1 Centre for Tropical Medicine and Global Health, Nuffield Department of Medicine, University of Oxford, Oxford, UK; 2 Programme for Emerging Infections, Infectious Disease Division, International Centre for Diarrheal Disease Research, Bangladesh, Dhaka, Bangladesh; 3 Pandemic Sciences Institute, Nuffield Department of Medicine, University of Oxford, Oxford, UK; 4 International Severe Acute Respiratory and Emerging Infection Consortium, University of Oxford, Oxford, UK; 5 Mahidol – Oxford Tropical Medicine Research Unit, Faculty of Tropical Medicine, Mahidol University, Bangkok, Thailand

**Keywords:** community engagement, Nipah virus, outbreaks, South Asia

## Abstract

The Nipah virus (NiV) infection is a highly fatal zoonotic disease with pandemic potential which has led to recurrent outbreaks in Bangladesh and India. While transmission pathways, including contaminated date palm sap and human-to-human spread, are increasingly identified, significant uncertainties remain. With no approved therapeutics or vaccines, prevention depends on addressing ecological and behavioural drivers of transmission. This viewpoint draws on selected evidence from NiV outbreaks and response to other zoonotic disease epidemics, such as Ebola, rabies, and the Marburg virus disease, we seek to foster discussion on why community engagement could be central to NiV prevention and preparedness. We highlight the relevance of community engagement through a spectrum of its intensity, which distinguishes between community-oriented, community-based, community-managed, and community-owned approaches. Adapting an existing model, we discuss how community engagement principles can be applied to tackle recurring NiV outbreaks in Bangladesh and India. By aligning interventions with sociocultural realities, community engagement can improve acceptability, enhance early detection, strengthen outbreak response, and support preparedness for future vaccine and therapeutic research. However, evidence specific to NiV remains limited and lessons from other diseases should be applied judiciously. In the absence of medical countermeasures, participatory, locally grounded approaches offer a sustainable pathway to reduce recurrent outbreaks and prevent future spillover events.

The Nipah virus (NiV) infection, an emerging global health threat with pandemic potential [1], is a highly fatal zoonotic virus for which Pteropus fruit bats act as the natural reservoir [2–4]. Transmission occurs through contact with infected animals, their bodily fluids, or consumption of contaminated food products, with human-to-human transmission also being possible [5].

Following its emergence in Malaysia in 1998, the NiV has caused recurring outbreaks in Bangladesh and India [6,7], where the consumption of raw date palm sap is the most consistently documented route of bat-to-human NiV transmission [8]. Fruit bats contaminate sap collection sites with saliva, urine, and faeces, creating a direct route for bat-to-human transmission when the sap is consumed fresh [9]. Human-to-human spread can occur, as well, particularly among close contacts and caregivers [10]. Evidence from India is more heterogeneous: early outbreaks in West Bengal in 2001 and 2007 were characterised by human-to-human and nosocomial transmission, with no confirmed epidemiological link to date palm sap consumption [11].

Since 2018, confirmed cases of outbreak in India have occurred in Kerala and West Bengal [12–14]. The recurring nature of the outbreaks in Kerala may reflect a combination of ecological change, increased human–bat interactions, and strengthened surveillance systems capable of identifying cases that may previously have gone undetected [13]. Environmental factors such as land-use change, habitat fragmentation, and human encroachment into bat habitats have also been proposed as contributors to spillover risk [14].

The NiV poses a uniquely urgent public health challenge, as prevention remains the primary response strategy due to a lack of established treatments and therapeutics [4,7,15–17]. Importantly, NiV transmission is not solely biological, but is also ecological and behavioural [8,18,19], with human activities, including consumption practices and environmental changes, likewise being closely linked to spillover events [8].

In this viewpoint, we argue that community engagement should be considered a core component of NiV preparedness and response. We build on an existing community engagement framework proposed by the World Health Organization which explains the intensity and nature of its approaches. This framework attempts to explain the community engagement strategies to tackle the recurrent NiV outbreaks in Bangladesh and India. We draw on published NiV literature and relevant lessons from other zoonotic disease responses where community participation, trust-building, and locally-led action contributed to outbreak prevention and control. While transferability of these approaches between diseases has limitations, these experiences can offer useful insights for strengthening NiV preparedness in settings where biomedical countermeasures remain limited.

With several vaccines and therapeutics under development, meaningful community engagement is essential [20–22] Involving affected communities, including patients and survivors, in research design and implementation helps ensure that interventions are relevant and acceptable. Engagement can inform practical aspects such as delivery methods and cultural acceptability, while also providing insights into local behaviours linked to disease transmission [23].

## COMMUNITY ENGAGEMENT AS A STRATEGY TO COMBAT SIMILAR DISEASES

Community engagement relates to how stakeholders (organisations, research groups, and individuals) work collaboratively on design, leadership, implementation, and evaluation of research or an intervention with at-risk communities to achieve a collective outcome [24]. It can thus decentralise power and bring interventions and strategies that are in line with communities’ social, cultural, and operational feasibilities [25,26].

While direct evidence on community engagement for NiV remains limited, lessons from other zoonotic and outbreak-prone diseases can provide useful insights, particularly in terms of how community engagement can operate such as in trust-building, risk communication, stigma reduction, community surveillance, and behaviour change (**Box 1**). Unlike diseases such as onchocerciasis or malaria, NiV outbreaks are sporadic, highly fatal, and characterised by substantial uncertainty regarding transmission risk. Community engagement strategies for NiV thus require disease-specific adaptation to local outbreak contexts, levels of risk perception, and existing trust in health systems.

Box 1 **Case studies of community engagement in containment and prevention of zoonotic diseases through community engagement.**

**Ebola (West Africa) – community-led containment measures**

Ebola, one of the deadliest and most complicated epidemics in recent history, is believed to have originated from bushmeat consumption and exhibits high human-to-human transmission [27]. An initial biomedical intervention for Ebola, introduced without community involvement or consideration of their social and cultural burial practices, led to community opposition [28]. Consistently, a scoping review showed the critical role of engaging community members to implement community-based initiatives for Ebola containment [26]. Community-based interventions undertook a pluralistic approach, including community surveillance, care centres, education and mobilisation, deployment of community health workers, and community-led sanitation initiatives. Specific activities involved radio campaigns, community meetings, training and education programs, mobile phone messaging, and broader social mobilisation efforts [26]. These approaches contributed to greater community involvement and acceptance leading to improved case detection, timely isolation and treatment, and ultimately, a reduction in pandemic spread. In Liberia, community engagement approaches led to smooth implementation of interventions such as safe burials. Some of these approaches included social mobilisation, engagement of community leaders, and case management under a community event-based monitoring programme, which played a key role in containing the spread of Ebola [26].

**Rabies (Burkina Faso) – rabies free Burkina Faso community engagement**

Rabies, another zoonotic disease, is a fatal, yet vaccine-preventable viral infection that affects the central nervous system. It is primarily transmitted to humans through bites, scratches, or contact with saliva from infected animals – most commonly dogs, which account for nearly 99% of human rabies cases worldwide [29]. An example of effective community engagement in rabies control comes from Burkina Faso, where public awareness about rabies prevention and treatment had previously been limited. The Rabies-free Burkina Faso initiative placed community engagement at the centre of its strategy to combat the disease. By involving diverse community groups – including children, parents, students, and professionals – the programme successfully addressed critical knowledge gaps regarding rabies transmission, the importance of dog vaccination, and the need for timely post-exposure prophylaxis. Information was disseminated through social media, radio and television interviews, school-based activities, and local seminars, ensuring that messages were accessible, context-specific, and widely distributed.

**Marburg virus disease (Ghana) – emergency preparedness by community engagement**

The Marburg virus disease is a severe zoonotic disease which, akin to NiV, is linked to exposure to bats. It spreads *via* direct contact with infected bodily fluids or contaminated materials, though early care can reduce its otherwise high fatality rate of 88% [30]. A qualitative study on emergency preparedness and response to Marburg virus disease in Ghana underscored the importance of adopting a bottom-up approach to address misinformation in resource-constrained settings during health crises [31]. Its findings suggest that effective preparedness for diseases similar to Marburg virus disease requires a comprehensive strategy that focuses on upskilling frontline workers, institutionalising mental health support mechanisms, strengthening patient engagement practices, and developing structured communication channels with health authorities. The study further suggests that faith-based organisations in particular should embed these measures within their operational frameworks to close existing gaps and enhance their capacity for robust risk communication and community engagement.

**Figure 1:**
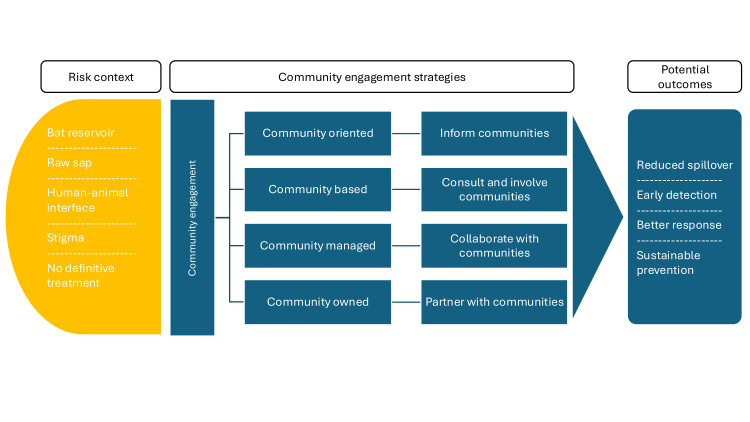
Conceptual framework for community engagement in Nipah virus prevention in Bangladesh and India. The framework illustrates the progression from risk context to community engagement strategies across increasing levels of participation and their corresponding mechanisms and potential public health outcomes.

To integrate these lessons, we adapt the World Health Organization’s community engagement continuum, which operates by a degree of community involvement in planning, implementation, leadership, and decision-making (**Figure 1**). For NiV prevention and preparedness, these levels can be conceptualised as community-oriented, community-based, community-managed, and community-owned approaches. Rather than representing a rigid sequence, these categories should be viewed as complementary and overlapping methods that may operate simultaneously depending on local needs, resources, and outbreak conditions.

### Community engagement strategies

The four engagement categories differ primarily in who leads activities, who makes decisions, and how responsibility is shared between communities and formal institutions. Clarifying these distinctions is important because many engagement activities can operate at multiple levels depending on how they are implemented [32].

#### Community-oriented

Community-oriented engagement focuses on ensuring that public health interventions are designed with an understanding of local beliefs, priorities, livelihoods, and sociocultural realities. At this level, communities inform intervention design, while decision-making remains largely led by health authorities and researchers [33].

Lessons from COVID-19 suggest that risk communication is most effective when messages are tailored to local contexts and delivered through trusted channels [34]. For NiV, this includes understanding community perceptions of bats, food practices, healthcare seeking, and disease-related stigma [35]. Community-oriented approaches should address behavioural and social factors associated with transmission risk, while recognising uncertainty surrounding some proposed pathways. They should also address stigma experienced by affected individuals, families, and survivors. Risk reduction efforts should recognise the cultural and economic significance of date palm sap collection and consumption, rather than portraying these practices as inherently problematic. Community leaders, religious leaders, teachers, local government representatives, sap collectors, and respected community members can all play important roles in facilitating dialogue and building trust.

Culturally-tailored health promotion campaigns may encourage practical risk reduction measures, including the use of protective barriers during sap collection and safer processing methods. However, evidence regarding the effectiveness, affordability, and long-term acceptability of these measures remains limited [8,36]. Public health messaging should, therefore, emphasise informed risk reduction, rather than blame or prohibition.

#### Community-based

Community-based engagement involves delivering interventions through existing community structures and trusted local actors. While strategic direction may remain with health authorities, implementation occurs within communities and relies on local participation, accessibility, and trust.

For NiV prevention, community-based approaches can strengthen awareness, surveillance, referral pathways, and early response activities [33]. In India, accredited social health activists, Anganwadi workers, and local primary healthcare teams already play important roles in community health promotion. In Bangladesh, community health care providers, health assistants, family welfare assistants, and community health workers employed by non-governmental organisations have extensive experience supporting disease prevention programmes.

These frontline workers can support community education, identify suspected cases, facilitate referral and contact tracing activities, and help address misinformation during outbreaks. Because they are embedded within communities, they may be particularly effective in communicating uncertainty and promoting trust in public health recommendations. Community-based approaches are also important for strengthening event-based surveillance. Community members often recognise unusual illnesses, animal deaths, or changes in local disease patterns before formal health systems. Creating mechanisms for reporting concerns through trusted local networks may improve early detection and outbreak response.

#### Community-managed

Community-managed engagement involves communities taking a leading role in organising, implementing, and overseeing engagement activities. At this level, local stakeholders move beyond participation and become active managers of interventions.

For NiV, this approach can build on successful community-led health initiatives previously implemented in Bangladesh and India. Religious leaders, local government representatives, teachers, youth groups, women’s organisations, and community volunteers may all contribute to the planning and delivery of engagement activities. Experiences from COVID-19 demonstrated that religious leaders can influence risk perception, trust, and acceptance of public health measures [37,38] Similar partnerships could support NiV preparedness by helping disseminate accurate information, addressing rumours, and reducing stigma.

Community theatre, folk media, school-based activities, and locally developed communication campaigns may also provide culturally relevant mechanisms for discussing transmission risks, symptoms, and preventive practices. Such approaches can encourage dialogue, rather than one-way communication and may be particularly valuable in areas where trust in formal institutions is limited. Importantly, community-managed approaches can help ensure that interventions remain responsive to local priorities and concerns. During outbreaks, community committees may support quarantine arrangements, coordinate assistance for affected households, and facilitate communication between communities and health authorities.

#### Community-owned

Community-owned engagement represents the highest level of participation, where communities exercise substantial decision-making authority over intervention priorities, implementation strategies, and local governance mechanisms. While examples of fully community-owned NiV prevention programmes remain limited, evidence from other public health initiatives demonstrates the potential value of community ownership in sustaining long-term behavioural and organisational change.

Experiences from community-directed interventions in Africa have demonstrated that communities can successfully lead complex public health programmes when provided with adequate resources, training, and institutional support [39]. However, important differences exist between neglected tropical disease programmes and NiV outbreak preparedness. Unlike routine preventive interventions, NiV outbreaks are unpredictable, infrequent, and characterised by high levels of uncertainty. Consequently, lessons from these programmes should be adapted, rather than adopted directly.

One example relevant to NiV is the promotion of bamboo skirt barriers covering date palm sap collection sites in Bangladesh. Pilot studies demonstrated that communities were willing to adopt these barriers under certain conditions, although evidence regarding long-term uptake and population-level impact remains limited [8,40]. These interventions nevertheless illustrate how locally acceptable risk reduction measures can emerge through collaboration with communities rather than through externally imposed directives.

### Operational recommendations for policy and practice

To strengthen preparedness for future outbreaks, community engagement should be embedded within national and subnational NiV preparedness plans. Specific actions may include:

- Ministries of health: integrate community engagement strategies into preparedness frameworks and outbreak response plans.
- Surveillance systems: strengthen community-based and event-based surveillance mechanisms to support early detection.
- Local governments: support community dialogue platforms and facilitate collaboration between health authorities and local stakeholders.
- Community health workers: receive training on NiV risk communication, stigma reduction, referral pathways, and outbreak reporting.
- Sap collectors and producers: participate in co-designed risk reduction initiatives that consider livelihoods, feasibility, and cultural practices.
- Schools and educational institutions: incorporate awareness activities that improve understanding of zoonotic disease risks.
- Religious and community leaders: support trusted communication channels and counter misinformation during outbreaks.
- Research teams and funding agencies: engage communities early in vaccine and therapeutic research planning, including consent processes, risk communication, participant protection, and dissemination of findings.
- Actively involve survivors and affected households in outbreak preparedness planning, risk communication, stigma reduction initiatives, and co-design of community engagement strategies.

## CONCLUSIONS

Preventing NiV outbreaks requires more than biomedical preparedness alone. In Bangladesh and India, opportunities for spillover and transmission are shaped by interactions among ecological change, livelihoods, cultural practices, health systems, and public trust. Consequently, community engagement should be considered a core component of outbreak preparedness and response rather than an adjunct activity.

Importantly, community engagement should not be viewed solely as a mechanism for promoting compliance with public health recommendations. Rather, it should create opportunities for communities to shape interventions, contribute local knowledge, identify feasible risk-reduction strategies, and participate in preparedness planning. Such approaches are particularly relevant for NiV, where uncertainty surrounding transmission pathways, the absence of widely available countermeasures, and the potential for fear and stigma can undermine outbreak response efforts.

## Data Availability

**Data availability:** No data were generated during the writing of this viewpoint.
